# Retention of people who inject drugs enrolled in a ‘medications for opioid use disorder’ (MOUD) programme in Uganda

**DOI:** 10.1186/s13722-024-00468-4

**Published:** 2024-05-15

**Authors:** Peter Mudiope, Brian Byamah Mutamba, Liz Komuhangi, Joan Nangendo, Stella Alamo, Bradley Mathers, Fredrick Makumbi, Rhoda Wanyenze

**Affiliations:** 1 Makerere University School of Public Health, Kampala, Uganda; 2 Butabika National Referral Mental Hospital, Kampala, Uganda; 3 Makerere University College of Health Sciences, Kampala, Uganda; 4 United States of America Centers for Disease Control and Prevention, Kampala, Uganda; 5 Global HIV, Hepatitis and Sexually Transmitted Infections Programmes, World Health Organization, Geneva, Switzerland; 6 Kirby Institute, University of New South Wales Sydney, Sydney, Australia

**Keywords:** Retention, People who inject drugs, Medication for opioid-use disorder, Uganda

## Abstract

**Background:**

Injection Drug use is associated with increased HIV risk behaviour that may result in the transmission of HIV and poor access to HIV prevention and treatment. In 2020, Uganda introduced the ‘medication for opioid use disorder (MOUD) treatment’ for People who inject drugs (PWID). We analysed the 12-month retention and associated factors among PWID enrolled on MOUD treatment in Kampala, Uganda.

**Methods:**

We conducted a retrospective analysis of 343 PWID with OUD who completed 14 days of methadone induction from September 2020 to July 2022. Retention was defined as the number of individuals still in the programme divided by the total number enrolled, computed at 3-, 6-, 9-, and 12 months using lifetable and Kaplan-Meier survival analyses. Cox proportional regression analyses were conducted to assess factors associated with retention in the programme in the first 12 months.

**Results:**

Overall, 243 (71%) of 343 participants stabilized at a methadone dose of 60 mg or more. The majority of participants were males (*n* = 284, 82.8%), and the median (interquartile range, IQR) age was 31 (26–38) years. Most participants (*n* = 276, 80.5%) lived 5 km or more away from the MOUD clinic. Thirty (8.8%) were HIV-positive, 52 (15.7%) had a major mental illness and 96 (27.9%) had a history of taking alcohol three months before enrollment. The cumulative retention significantly declined from 83.4% (95%CI = 79.0–87.0) at 3months to 71.9% (95%CI = 67.2–76.6) at 6months, 64% 95%CI = 58.7–68.9) at 9months, and 55.2%; 95% CI (49.8–60.3% at 12months. The 12-month retention was significantly higher for participants on methadone doses of 60 mg or more (adj.HR = 2.1, 95%CI = 1.41–3.22), while participants resident within 5 km of the MOUD clinic were 4.9 times more likely to be retained at 12 months, compared to those residing 5 km or more, (adj. HR = 4.81, 95%CI = 1.54-15). Other factors, including predisposing, need, and enabling factors, were not associated with retention.

**Conclusion:**

Our study demonstrates acceptable 12-month retention rates for people who inject drugs, comparable to previous studies done in both developing and developed countries. Sustaining and improving retention may require enhanced scaling up of MOUD dose to an optimal level in the first 14 days and reducing the distance between participant locale and MOUD clinics.

**Supplementary Information:**

The online version contains supplementary material available at 10.1186/s13722-024-00468-4.

## Background

Opioid use disorder is associated with physiological, behavioural, and social consequences such as premature mortality, criminality, violence, and suicide [1, 2]. Additionally, injecting drug use is associated with an increased occurrence of drug overdose, risk of HIV and Hepatitis C infections and a key driver of the HIV epidemic [1, 3–5]. Further, people who inject drugs (PWID) experience stigma and social exclusion that hinder them from accessing social services and healthcare [6, 7]. The use of opioid agonists such as methadone and buprenorphine as a long-term maintenance treatment is effective in reducing drug use and its HIV-related risk among people who inject drugs (PWIDs) [3, 8–10]. Medication for Opioid use disorder (MOUD) is recommended by the World Health Organization (WHO), the United Nations Office of Drug Control (UNODC), and the Joint United Nations Programme on HIV/AIDS (UNAIDS) as an essential harm-reduction intervention for PWID [11]. MOUD, when effectively taken reduces the risk of transmitting HIV and viral hepatitis, by substituting the injectable heroin with oral methadone or buprenorphine. Heroin users then switch from the “black market” to legally dispensed medicines under the care of a health professional, therefore minimizing the risk of overdose and other medical complications. However, despite the availability of the WHO/UNODC guidelines, few developing countries have taken up MOUD programmes [12, 13]. The factors contributing to low uptake and subsequent client retention in the MOUD vary and are not thoroughly understood in such environments [14–16].

In Uganda, the national harm reduction guidelines (first developed in 2019), provide for the nine harm reduction interventions as adopted from the WHO/UNODC harm reduction guidelines [11, 17]. Before the development of these guidelines, the country had successfully piloted a needle and syringe programme(NSP), with follow on donor-supported projects in Kampala and Mbale cities [18]. In addition, few private rehabilitation facilities provide very expensive abstinence-based addiction management services around Kampala. However, in September 2020, the first OUD treatment clinic was set up at Butabika National Referral Mental Hospital, to provide comprehensive harm reduction package for PWIDs. At the clinic, oral methadone has been the preferred medication used for addiction management. Following lessons learned, Uganda with support from PEPFAR (country operational Plan 23–25) and Global fund cycle 7, planned to roll out OUD treatment to one additional site in 2024. We assessed retention and associated factors for retention among opioid drug users followed up in the nascent MOUD programme in Kampala, Uganda.

## Methods

We conducted a retrospective cohort analysis of 343 people who inject opioid drugs, enrolled in the nascent MOUD programme and attained a maintenance methadone dose, during September 2020 and July 2022.

### Study setting

The MOUD programme commenced in September 2020 at Butabika National Referral Mental Hospital in Uganda’s capital city, Kampala, as a learning site to inform programme scale-up. The MOUD clinic, located in the alcohol and drug unit, has a separate access entrance used by only MOUD clients, and staff. The separate entrance allows MOUD clients to access the clinic with minimal security checkpoints versus what other clients have to go through to access other services at the hospital; minimizes the chance of inpatients (under rehabilitation treatment for alcohol use and other drug-related problems) accessing illicit drugs through the MOUD clients; and improves retention on long in-patient care.

In addition to offering tertiary mental health services, Butabika National Referral Mental Hospital also provides integrated primary health care (PHC) services such as HIV, TB and STI diagnosis and treatment. This one-stop services integration model enables the hospital to offer a person-centered delivery model.

### Participants

The Uganda Harm Reduction Network (UHRN), a community-based organisation, provided information to PWID community members on the availability of MOUD services and referred interested and eligible participants to the clinic for enrollment. The screening process involved establishing the participant’s opioid use disorder (OUD) status. The diagnosis of OUD was made based on the Diagnostic and Statistical Manual of Mental Disorders [19]. The screening/preparation process also involved establishing the participant’s interest in enrolling in the programme; education on harm reduction; social support; determining the acceptability of MOUD; baseline assessment of clinical and mental health status, HIV sero-status, hepatitis C status, urine toxicology, and Clinical Opiate Withdrawal Scale (COWS) score at enrolment [20, 21]; and developing a treatment plan. Participants 18 years and above with confirmed opioid use disorder, a history of injecting drug use in the last 3 months, were willing to adhere to the clinic dos and don’ts, and provided written consent to enroll on MOUD were eligible and accepted in the study. Participants with coexisting severe alcohol use disorder, severe liver disease or chronic pain were referred for appropriate management. To understand the relationship between the maintenance methadone dose and retention, we analyzed only participants who attained stable dose at 14 days. The 14-day time point is important because, at this point, unlike during the induction phase, OUD patients are stable and are expected to be receiving a maintenance dose with full benefits of methadone, where the psychosocial problems inherent in opiate addiction are relieved upon the methadone maintenance. Studies by Kling MA et al. (2000) and Gavin Bart (2012 separately demonstrated that during stabilization, methadone binds to approximately 30% of mu-opioid receptors, thereby allowing the remaining receptors to carry out their typical physiological functions in pain, reward, and mood modulation [9, 22].

### The intervention

The medication used for MOUD is oral methadone provided as a daily observed dose. Each day participants were received by a peer at the clinic reception for triage, to receive daily methadone dose, visit a counsellor/psychologist or clinician. The decision of what services a participant received was guided by the presenting complaints, including the need for clinical review, dose review, PrEP, or other medication refills and summarised in the treatment plan.

### Induction and follow-up and termination

The MOUD initiation involved starting patients on a minimum safe dose of methadone that reduces craving or withdrawal symptoms, carefully increasing this dose to reach a maintenance dose over a period of 14 days. Participants with signs of opioid intoxication or sedation had to abstain from drug use for at least six hours before induction. The initial induction dose was 10 mg methadone, adjusted upward on a 5 mg scale until stabilization was reached. The dose was adjusted upward or downward based on participant needs, preferences, and clinic attendance. For example, participants who missed more than three consecutive days of clinic attendance, had their methadone dose reduced based on clinical assessment. The participants came to the clinic daily for observed methadone dosing. The participants were also required to visit the clinician and or counsellor every three months for physical, mental, social, and or treatment plan review. Participants were required to attend weekly group education sessions at both the MOUD clinic and the referring community drop-in-centre. Counsellors and peers provided individual and group counselling, education sessions, and psychosocial support services. Peers used their lived experiences with drug use to effectively deliver on their role of counselling and educating their clients and communities.

### Cessation and management of missed doses

Close dose monitoring for treatment compliance was performed using an automated methadone dispenser that provided a daily print-out list of participants who missed their doses. Because a clinically significant loss of tolerance to opioids may occur within as little as three days without methadone, the participant’s dose was adjusted if they missed three or more consecutive doses and rapidly increased once the response to the lower dose was assessed.

Participants were supported to voluntarily terminate their participation through counselling and a stepwise down titration of methadone, and this was viewed as self-cessation. Participants who violated the programme conditions(as described in the consent form) were involuntarily terminated. A participant was declared not retained if they missed the methadone dose for 30 consecutive doses. In this evaluation, participants who returned after being declared not retained, underwent medical and psychosocial assessment and were advised to restart methadone on meeting the enrollment criteria, as a new participant.

### Measurements

Baseline variables recorded during enrollment included sex, age, housing status (assigned as stable or unstable based on whether the participant had a place of abode, easy to locate by the peer and had stayed in the area for at least one month and was not planning to shift to a place outside MUOD programme catchment areas in the next three months). Other variables included distance between residence and MOUD, alcohol use based on audit tool [23], imprisonment during the previous 3 months, HIV status assessed following the Uganda national testing algorithm, mental illness and COWS score. At follow-up visits, any change in a participant’s residence was recorded. The daily dose of methadone was recorded using a Meta-measure dispenser [24]. Urine drug screening (UDS) using enzyme immune assay based rapid test strips, was done at enrollment and randomly during follow-up to guide the tailoring of individualized intervention toward relapse prevention.

In this evaluation, the main outcome, one-year retention, was determined by collecting data on each participant who received methadone from the MOUD clinic reached maintenance dose at 14 days, and followed for 12 months.

### Data management

Data extraction followed the data management protocols at the clinic. For methadone daily dosing, data were electronically extracted from the meta-measure system by the pharmacist. The MOUD clinic staff trained on the protocol, conducted a participant chart review, and extracted data on the demographic, social, clinical and psychiatric characteristics. The data was reviewed by the first and second author before it was keyed into an open data kit database. The different data sets were saved in a comma-separated version (CSV) delimited format and imported into Stata version. 14.2 (College Station, Texas) for cleaning and analysis.

### Data analysis

The analysis includes participants who reached 14 days of follow-up, a point after which a participant was presumed to be at a stable methadone maintenance dose (the primary independent variable examined. Baseline data were summarised using medians and interquartile ranges for numerical variables and proportions for categorical variables. For each participant, the median daily methadone dose was computed for all doses taken after the initial 14 days when a participant was on a maintenance dose. The median dose was a better estimate measure of methadone exposure compared to the mean doses because methadone dose data were not normally distributed.

The median dose was then categorized as a low dose equivalent to less than 60 mg, and a high dose equivalent to 60 mg and above [11, 25]. The variations in participants’ characteristics based on the main predictor dose of methadone (low versus high dose) were assessed using the Pearson Chi square test. The number of days from the date of enrollment until the date the participant was lost from care, died, discharged, or until the end of follow-up was taken to calculate retention duration in treatment using the Kaplan‒Meier method. The life table method was used to calculate the cumulative retention rate. The clients discharged from the programme after successful cessation of drug injection or who remained in the programme 12 months from the enrollment date were considered retained. However, participants who died or were lost from care were classified as failing(non-retention). Participants enrolled in MOUD who dropped out during the first 14 days were excluded from further analysis because these had not attained the optimal maintenance dose, the main predictor in this analysis. Using Cox’s proportional hazards model, we determined the factors associated with retention on MOUD. Independent variables were enrolled in bivariate analyses, and variables significantly associated with retention (p-value < 0.1) and or known to have clinical association with retention in the MOUD programme, were included in the Cox regression multivariate analyses. Hazard ratios (HRs) and 95% confidence intervals are estimated. All independent variables were transformed into categorical variables. All analyses were performed using Stata version. 14.2 (College Station, Texas).

## Results

Overall, 386 records of injecting drug users enrolled in the MOUD programme from September 2020 to July 30, 2022, were reviewed. Forty-three participants did not complete the induction phase(first 14 days) and were excluded from this analysis because they had not attained a maintenance dose of methadone. We analyzed data from 343 participants. These were predominantly male (*n* = 284, 82.8%), with a median (interquartile range, IQR) age of 31 [26–38] years. 240 (69.9%) participants had a stable housing status (residence). The majority were living 5 km or more away from the MOUD clinic (*n* = 276, 80.5%) away from the MOUD clinic, and 53 (15.4%) relocated to places near the clinic during the 12 months after enrollment. Thirty (8.8%) were recorded as HIV-positive, 54 (15.7%) had a major mental illness, and 96 (27.9%) had a history of taking alcohol three months before enrollment. On average, 20 (5.8%), 167 (48.7%), and 119 (34.7%) had severe, moderate, and mild COWS scores, respectively. The participants who took a low methadone maintenance dose (less than 60 mg daily) were not different from those who took a high daily dose methadone(60 mg or more) treatment regarding their baseline characteristics (Table 1).

**Table 1:** Characteristics of participants enrolled in Medication for Opioid Use Disorder programme in Kampala, Uganda(September 2020-July 2022): Stratified by methadone dosage

Variable	Total (%) (*N* = 343)	Methadone dose < 60 mg (*n* = 243)	Methadone dose > = 60 mg (*n* = 100)	Chi2 *P* value
**Age**: median (IQR)	31 (26–38)	31(26–38)	31(25–38)	
**Sex** Male	284 (82.8)	202(83.3)	82 (82)	0.801
Female	59 (17.2)	41 (16.9)	22 (18)	
**Distance**: less than 5Km	67(19.5)	45(18.2)	22 (22)	0.46
5Km and more	276 (80.5)	198 (81.5)	78 (78)	
**Relocated close to MOUD clinic**				
YES	53 (15.4)	34 (14)	19 (19)	0.244
NO	290 (84.6)	209 (86)	81 (81)	
**Missed doses**: None	168(48.9)	114(46.9)	54(54)	0.233
>=1 missed dose	176(51.1)	129(53.1)	46(46)	
**Alcohol use last 3 months**				
YES	96 (27.9)	69 (28.4)	27 (27)	0.925
NO	247 (72.1)	174(71.6)	73(73)	
**HIV status**: Positive	30 (8.8)	22 (9.1)	8 (8)	0.754
Negative/Unknown	313 (91.2)	221 (90.9)	90 (92)	
**Mental Health Illness**				
YES	54 (15.7)	37 (15.2)	17 (17)	0.682
NO	289 (84.3)	206 (84.8)	83 (83)	
**Housing status**: unstable	103 (30.1)	74(30.4)	29 (29)	0.433
Stable	240 (69.9)	169 (69.6)	71 (71)	
**COWS Score**: Mild	119 (34.7)	86 (35.4)	33 (33)	0.539
Moderate	167 (48.7)	113 (46.5)	54 54)	
Severe	20 (5.8)	16 (6.6)	4 (4)	
Missing	37 (10.8)	28 (11.5)	9 (9)	
**Imprisonment in last 3 months**				
YES	15 (4.4)	10 (4.1)	5 (5)	0.312
NO	204 (59.5)	139 (57.2)	65(65)	
No response	124 (36.1)	94 (38.7)	30 (30)	

**Table 2:** Factors associated with 12 months retention of individuals enrolled in the Medication for Opioid Use Disorder programme in Kampala, Uganda(September 2020-July 2022)

Variables	Retention percentage (95%CI)	Unadjusted HR(95%CI)	Adjusted HR(95%CI)
**Overall retention**	55.6 (50.3–60.6)		
**Dose of methadone**			
Low (< 60 mg)	48.4(41.9–54.5)	1	1
High ( > = 60 mg)	71.8 ((61.8–79.6)	2.19(1.45–3.30)	2.1(1.41–3.22)
**Age**: 15-24yrs	57.4(44.3–68.5)	1	
25-34yrs	50.3(42.6–57.6)	1.12(0.73–1.71)	
Above35yrs	61.5(51.7–69.9)	0.88(0.54–1.43)	
**Sex** Male	56.9(50.9–62.4)	1	
Female	46.8(33.6–58.9)	0.71(0.48–1.05)	
**Distance**: More or = 5Km	47.2(41.2–52.9)	1	1
Less than5Km	88.1(77.5–93.8)	6.01(2.95–12.26)	4.81(1.54-15.0)
**Relocated close to MOUD clinic**			
**NO**	49.1 (43.2–54.7)	1	1
YES	88.7 (76.5–94.8)	6(2.65–13.58)	0.68(0.19–2.42)
**Missed doses**: None missed	63.7(55.9–70.5)	1	1
One/more doses	47.2(39.6–54.3)	0.69(0.49–0.95)	0.85(0.61–1.17)
**History of Alcohol**			
NO/No response	58.1 (51.7–64)	1	
YES	47.4(37.1–57.1)	0.75(0.53–1.03)	
**HIV serostatus**			
POSITIVE	56.7(37.3–72.1)	1	1
NEGATIVE/UNKNOWN	55(49.3–60.3)	0.99(0.57–1.77)	0.98(0.55–1.74)
**Major Mental Health Illness**			
NO	56.1(50.2–61.6)	1	1
YES	50(36.1–62.4)	1.18(0.78–1.79)	1.1(0.71–1.7)
**Housing** -Stable	55.5(48.9–61.5)	1	
Non-Stable	54.4(44.3–63.4)	0.93(0.66–1.3)	
**COWS Score**	50(40.8–58.7)	1	
MILD			
MODERATE	60.3(52.5–67.3)	1.33(0.93–1.88)	
SEVERE	42.8(21.9–62.3)	0.85(0.43–1.75)	
**Incarceration history**			
YES	40(16.5–62.8)	1	
NO	55.7(48.6–62.2)	1.37(0.71–2.94)	

### Retention

**Fig. 1:**
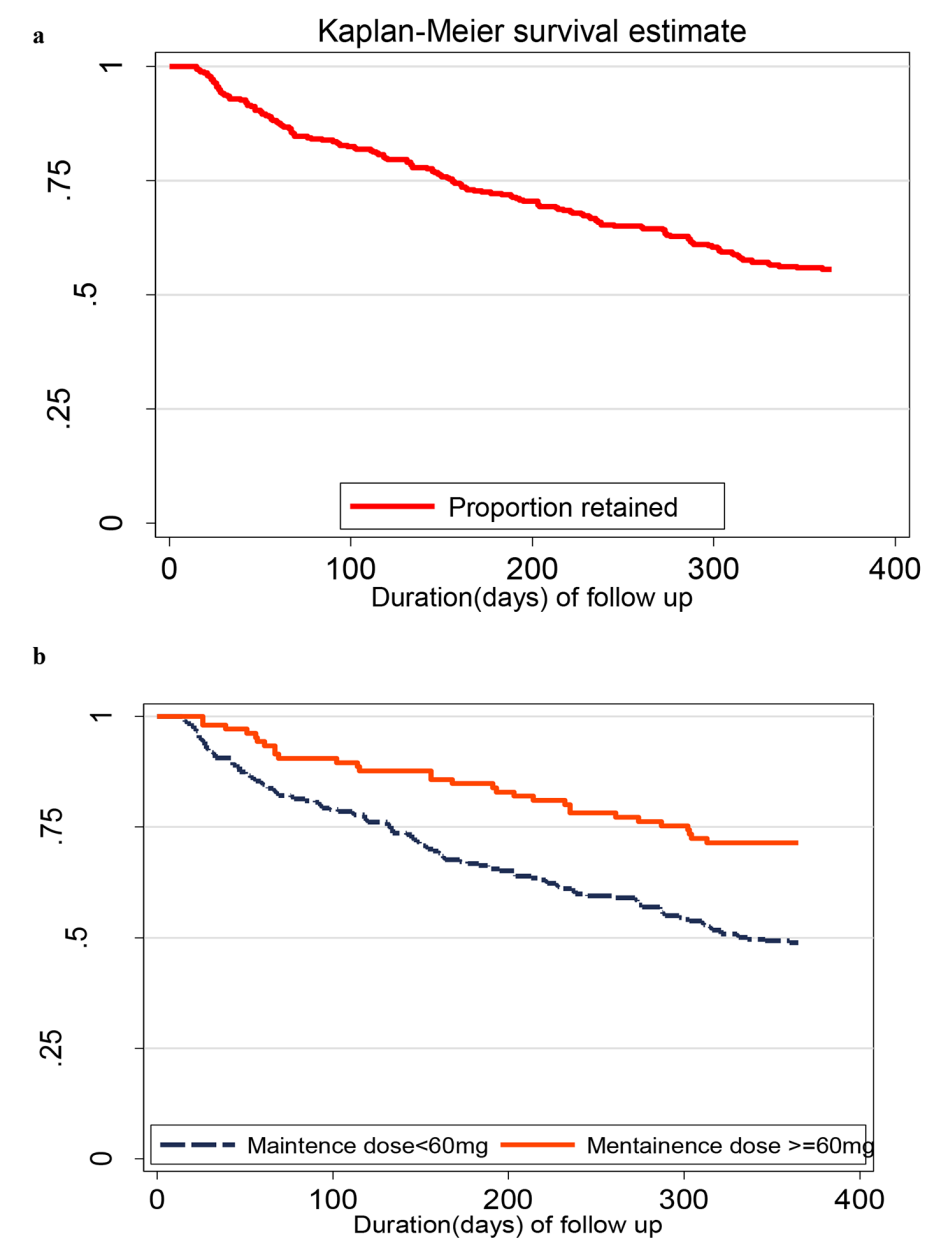
Proportion of participants retained in the MOUD programme in Kampala, Uganda (September 2020-July 2022)

Overall, 186 of 343 participants remained in the programme through 12 months, yielding a retention rate of 55.2% [95% confidence interval (CI), 49.8–60.3%]. Retention declined over the 12months, 83.4% (95% CI = 79.0–87.0) at 3 months, 71.9% (95% CI = 67.2–76.6) at 6 months and 64% 95% CI = 58.7–68.9) at 9months after enrollment (Fig. 1a).

The overall 12 months retention was higher at 71.8%, 95% CI (61.8–79.6%) in participants who took methadone doses higher than or equal to 60 mg/day, compared to 48.4%, 95% CI (41.9–54.5%) for those who took doses less than 60 mg/day(Fig. 1b). More participants (88.1% [1], 77.5–93.8%) who were living within a 5 km radius compared to (48% [1], 42.1–53.6%) of those who lived more than 5 km away from the clinic completed 12 months in the programme. There were, however, no differences in the retention of participants based on their demographic, social, clinical, and psychiatric characteristics (Table 2).

### Reasons for non-retention at 12 months

Among the 157 participants who did not complete 12 months, three (1.9%) had died, 30 (19.7%) voluntarily terminated from the programme, five (3.2%) voluntarily terminated after quitting drug injecting, 117 (74.5%) missed clinic attendance for more than thirty days and were not reachable by programme staff, one participant was involuntarily terminated due to confirmed consistent diversion of methadone, and one was admitted to a private rehabilitation facility.

### Factors associated with retention

At bivariate, individual factors associated with 12months retention include methadone maintenance dose, distance from the clinic, and adherence status. Participants taking a methadone maintenance dose of 60 mg or more were two times more likely to reach 12 months retention than those who took a methadone dose less than 60 mg (HR = 2.19, 95% CI = 1.45–3.30). The participants living within a 5 km radius were six times more likely to be retained in the programme compared to those living more than 5 km away from the clinic (HR = 6.01, 95% CI = 2.95–12.26). Equally, participants who relocated to locations closer to the clinic during the study period were likely to be retained in the programme compared to those who did not relocate (HR = 2, 95% CI = 2.65–13.58). Participants who regularly attended the clinic and took all their daily doses were more likely to be retained in the study compared to their counterparts who missed at least one or more clinic days. We did not find any statistical association between retention and other factors studied, including participants’ age, sex, HIV status, mental health status, housing status, and incarceration history. In the multivariate analysis, a methadone maintenance dose of 60 mg or more was persistently associated with 12 months of retention (HR = 2.1, 95% CI = 1.41–3.22). For distance, participants living within a 5 km radius from the clinic were almost 5 times more likely to reach 12 months retention.

## Discussion

The study assessed medication opioid use disorder treatment retention and the factors that affect retention in an observed and structured methadone maintenance therapy programme in Uganda. More than half of PWID initiating MOUD and attaining stable doses within 14 days stayed on treatment for at least 12 months. Twelve-month retention was chosen because it has previously been described as a measure of treatment exposure that predicts positive treatment outcomes as well [26].

### Retention

The 55.5% retention found in our study varies when compared to that found elsewhere in developing countries. In South Africa, 81% of the participants were still in the study at 6 months, compared to the 72.2% reported in this study [15]. In Iran, retention was much lower, ranging from 15.8 to 34% at 12 months [14, 16]. Unlike the study in Iran, the high retention in our study may be attributed to having a voluntary admission of participants and presence of integrated management of concurrent physical health and psychiatric co-morbidities. The shift from a prohibition to a rights-centered approach to harm reduction, under Uganda Harm Reduction Guidelines 2019 [17], may have also contributed to higher retention of PWID in the programme. In a systematic review of programme reviews and studies performed in low- and middle-income areas, 12-month retention was found to average 56% (range 46–68%) [27]. Our results provide further evidence that the retention of the majority of MOUD participants can be achieved and sustained in low-income countries, especially if participants receive appropriate methadone maintenance doses within the first 14 days of enrollment. Despite programme and population variations, our retention is comparable to that in developed countries averaging between 40 and 50% [28–30].

### Factors associated with retention

Both Methadone doses greater than 60 mg/day, and a distance of less than 5 km from the MOUD clinic were significant predictors of 12 months of retention in the MOUD programme.

The finding that a median daily maintenance methadone dose greater than 59 mg resulted in better treatment retention provides additional evidence from low-income settings to strengthen the WHO recommendation on methadone [11] dosing and is consistent with previous studies [31, 32] from developed countries. The WHO guidelines recommend a methadone maintenance dose of 60-120 mg/day for optimal treatment outcome [11]. These findings are a reassurance to the conservative service providers in low-income countries such as Uganda, to prescribe high optimal doses of methadone at the earliest time possible based on participants’ needs.

The other significant predictor of retention in this study was distance to the clinic. The participants who stayed less than 5 km from the MOUD clinic were close to 5-times more likely to complete 12 months in the programme. Solmaz Amiri et al. reported comparable findings in their study done in Spokane County Washington, USA [33]. The MOUD clinic is based at the Butabika National Referral Mental Hospital, which is distant from most drug-injecting hotspots. Some participants would miss clinic attendances because they were not able to walk or failed to secure fees for transport to the clinic. To cut costs incurred for daily attendance, some participants had to relocate their residences to places near the clinic. Indeed, nine in 10 of those who relocated to nearby places were still in the programme 12 months after enrollment. These findings suggest that besides addressing the legal restrictions, stigma, and discrimination [3, 34], programmes should be located closer to PWID dwelling places to harness longer retention of participants Innovations from previous studies employing take-home doses, use of mobile units to dispatch medicines to hotspots, and community MOUD programmes [4] demonstrated improved retention of participants on MOUD. These results further support Uganda’s efforts to operationalize the mobile van that will decentralize methadone dispensing at public health facilities located close to hotspots of the people who inject drugs.

Perhaps, what remains unclear is how predisposing factors such as age and sex affect retention. In their study, Ball, and Ross (1991) demonstrated that young participants were more likely to be retained in treatment programmes; however, other studies found better retention for older participants [30, 35–38]. In this study, we found no association between age, sex and 12 months of retention in the MOUD programme. Equally, there was no association between retention and other predisposing factors (alcohol and other drug use), enabling factors (housing status), or need factors (mental illness, HIV status, COWS score, and incarceration) at enrollment. Previously, there was varying evidence of the association between age and retention.

### Limitations

Based on the behavioural model of health care utilization [39, 40], this study examined patient-centred predisposing and need factors associated with retention. The study did not cover the participant’s perception of treatment and provider-related variables. Participants may also have received other variable support from the CBOs, and these data were not reported in the existing facility records. Studying all participants who accessed MOUD services during the study period helped to minimize the occurrence of selection bias. However, this analysis successfully demonstrated acceptable retention rates, which could be improved further by prescribing high doses and decentralising the MOUD clinic closer to the PWID locale.

## Conclusion

Our study demonstrates acceptable 12months retention rates, especially among participants who took higher methadone doses, that were comparable to those recommended by WHO and reported in studies performed elsewhere in other countries. Sustaining and improving retention may require scaling up the MOUD dose to the optimal level in the first 14 days, and reducing the distance between the PWID participant locale and MOUD clinics through the adoption of differentiated service models such as mobile vans or hub and spoke models for MOUD dispensing. Further research using large samples and focusing on the provider-related factors and behavioural characteristics of participants may shed more insight into retention rates in MOUD programmes in low-income countries.

### Electronic supplementary material

Below is the link to the electronic supplementary material.

Supplementary Material 1

Supplementary Material 2

## Data Availability

The datasets analysed during the current study will be shared on reasonable request by the corresponding author.

## Abbreviations

CBO: Community-based organization
COWS: Clinical Opioid Withdrawal score
MOUD: medication for opioid use disorder
OUD: Opioid use disorder
PWID: People Who inject drugs
PWUD: People who use drugs
UDS: Urine drug screening
and UHRN: Uganda Harm Reduction Network Organization
