# Supplementary material for: Retention of people who inject drugs enrolled in a ‘medications for opioid use disorder’ (MOUD) programme in Uganda
Source: Addict Sci Clin Pract. 2024 May 15;19:39. doi: 10.1186/s13722-024-00468-4 (PMC11094991; doi:10.1186/s13722-024-00468-4)
Supplement: Supplementary file 1 — Supplementary Material 1 [file 13722_2024_468_MOESM1_ESM.docx]

**Responses to the issues raised by the Reviewers and the editor, indicating which changes were made**

**Title:** **: RETENTION OF** **PEOPLE WHO INJECT DRUGS ENROLLED IN A ‘MEDICATIONS FOR OPIOID USE DISORDER’ (MOUD) PROGRAMME IN UGANDA (Manuscript submitted to Addiction Science & Clinical Practice - ASCP-D-23-00156)**

**Comment 1:** Thank you for your revisions. Please attend to the below final revisions to your manuscript prior to publication.

**Authors’ Responses:** We sincerely appreciate this continued and thorough feedback that reflects the journal’s principle of scientific rigor

**Comment 2:** In the R2 version the authors included a rationale in the methods for why this is the appropriate way to conduct the study: ……I disagree that it is “more appropriate." Please delete the last two sentences of the above: "Consequently, it is now more appropriate to focus on studying modifiable predictors of retention beyond this stage. By identifying and addressing factors that influence treatment retention during this stage, healthcare providers can optimize the effectiveness of MUOD interventions and enhance long-term outcomes for individuals seeking recovery from opioid addiction."

**Authors’ Responses:** We concur with this recommendation of deleting the two sentences from lines 125 to 129. The subsection on participants now ends with the words “….reward, and mood modulation(9, 22)” See highlight in yellow on line 125

**Comment 3:** Throughout the paper it is now clear that the analyses were conducted on the cohort of people who completed 14 days, so I do not think the paper is misleading. I would still prefer that the authors conduct the survival analysis using the 386 initiators as the denominator, rather than the 343 14-day completers as the denominator. However, because they are transparent about their approach it is acceptable to me as it is. The rationale they provide is sufficient, but does not justify saying it is “more appropriate.”

**Authors’ Responses:** This observation is well-noted

**Comment 4:** 2.        Results: Reasons for non-retention at 12 months
In the Methods, the authors describe reasons for termination – self-cessation where patients voluntarily tapered off methadone with the support of their counselors, involuntary termination where they violated rules, missed 30 days where patients missed 30 consecutive days.  In the Results, the authors use slightly different terms – voluntarily withdrew, drug injection cessation, and admitted to private rehab facility. For missed 30 days and involuntary termination, the terms are consistent.

Please use consistent terms between the Methods and the Results.  Is voluntarily withdrew the same as self-cessation?

**Authors’ Responses:** This is noted. For consistency, we have replaced the word voluntarily withdrew with voluntary cessation in the results section. see Line 246-247 highlighted in yellow

**Comment 5:** What does drug injection cessation mean and how did you assess whether participants ceased or continued drug injection?

**Authors’ Responses:** At the OUD clinic, drug injecting status is systematically assessed during scheduled three-monthly client evaluation visits. A client is considered to have achieved drug injection cessation when they self-report abstinence from drug injections and demonstrate two consecutive negative urine drug screening tests, conducted at least three months apart. This dual verification approach enhances the reliability and validity of identifying successful drug injection cessation among our clients." Although MOUD down tapering for cessation commences any time clients request,

Clients who report having stopped injecting drugs remain on MOUD until they receive a second negative urine drug screening test result. However, MOUD down-tapering for cessation may commence at any time upon a client's request.

**Additional minor items:**
**Comment 6:** Line 147 – Instead of “Participants were needed…” replace with “Participants were required…”

**Authors’ Responses:** This comment is well noted. We have affected the recommendation. In line 143, the phrase “Participants were needed…” has been replaced with “Participants were required…” see yellow highlight

**Discussion**

**Comment 7:** Line 316 Change for clarity: These findings suggest that besides addressing the legal restrictions, stigma, and discrimination(3, 34), programmes should be located closer to PWID dwelling places to harness longer retention of participants.

**Authors’ Responses:** We agree with this recommendation. The change has been effected in lines 314 to 315 in yellow highlight

**Comment 8:** Figure 1: Re-label “survivor function” as “Proportion retained”

**Authors’ Responses:** This advise is well noted. We have replaced “survivor function” with “Proportion retained” in Figure 1

**Comment 9:** Figure 1a: Re-label “meddosecat = low” as “Maintenance dose<60mg” and Re-label “meddosecat = high” as “Maintenance dose ≥60mg”

**Authors’ Responses:** This advise is well noted. We have effected the recommendation and replaced meddosecat = high” with “Maintenance dose ≥60mg” in figure 1a
