# Supplementary material for: Retention of people who inject drugs enrolled in a ‘medications for opioid use disorder’ (MOUD) programme in Uganda
Source: Addict Sci Clin Pract. 2024 May 15;19:39. doi: 10.1186/s13722-024-00468-4 (PMC11094991; doi:10.1186/s13722-024-00468-4)
Supplement: Supplementary file 2 — Supplementary Material 2 [file 13722_2024_468_MOESM2_ESM.doc]

**The Chief Editor**

Addiction Science & Clinical Practice,

24th April 2024

Dear Editor,

**Subject: Submission of the Revised Manuscript - Retention of People Who Inject Drugs Enrolled in a ‘Medications for Opioid Use Disorder’ (MOUD) Programme in Uganda (ASCP-D-23-00156)**

We would like to express our sincere gratitude for accepting our manuscript for publication in Addiction Science & Clinical Practice. We also extend our thanks to you and the reviewers for their constructive feedback, which has greatly contributed to improving the quality and rigor of our work.

In response to the reviewers' comments, we have revised the manuscript, addressing each point in detail. The revisions are highlighted in yellow within the revised manuscript submitted via the submission system.

We firmly believe that the findings presented in our manuscript will make a valuable contribution to the enhancement of MOUD programs in Uganda and other similar settings. We are confident that the broad readership of your journal will facilitate the dissemination of our research to a diverse and engaged audience.

All the Authors have read and concurred with the contents of the revised manuscript, and there is no undisclosed conflict of interest to this paper.

We appreciate your consideration of our manuscript and look forward to hearing from you soon.

Thank you for your time and attention.

Yours sincerely,

**Dr. Peter Mudiope (MBchB, MSC. CEB, PhD fellow)**

P.O.Box 37757 Kampala, Uganda

Tel (+256-772864340)

Email: [pmudiope@gmail.com](mailto:pmudiope@gmail.com)
